# High-throughput screening of 2D van der Waals crystals with plastic deformability

**DOI:** 10.1038/s41467-022-35229-x

**Published:** 2022-12-05

**Authors:** Zhiqiang Gao, Tian-Ran Wei, Tingting Deng, Pengfei Qiu, Wei Xu, Yuecun Wang, Lidong Chen, Xun Shi

**Affiliations:** 1 State Key Laboratory of High Performance Ceramics and Superfine Microstructure, Shanghai Institute of Ceramics, Chinese Academy of Sciences, Shanghai, 200050 China; 2 School of Physical Science and Technology, ShanghaiTech University, Shanghai, 201210 China; 3 State Key Laboratory of Metal Matrix Composites, School of Materials Science and Engineering, Shanghai Jiao Tong University, Shanghai, 200240 China; 4 School of Chemistry and Materials Science, Hangzhou Institute for Advanced Study, University of Chinese Academy of Sciences, Hangzhou, 310024 China; 5 Center of Materials Science and Optoelectronics Engineering, University of Chinese Academy of Sciences, Beijing, 100049 China; 6 Center for Advancing Materials Performance from the Nanoscale (CAMP-Nano) & Hysitron Applied Research Center in China (HARCC), State Key Laboratory for Mechanical Behavior of Materials, Xi’an Jiaotong University, Xi’an, 710049 China

**Keywords:** Mechanical properties, Semiconductors

## Abstract

Inorganic semiconductors exhibit multifarious physical properties, but they are prevailingly brittle, impeding their application in flexible and hetero-shaped electronics. The exceptional plasticity discovered in InSe crystal indicates the existence of abundant plastically deformable two-dimensional van der Waals (2D vdW) materials, but the conventional trial-and-error method is too time-consuming and costly. Here we report on the discovery of tens of potential 2D chalcogenide crystals with plastic deformability using a nearly automated and efficient high-throughput screening methodology. Seven candidates e.g., famous MoS_2_, GaSe, and SnSe_2_ 2D materials are carefully verified to show largely anisotropic plastic deformations, which are contributed by both interlayer and cross-layer slips involving continuous breaking and reconstruction of chemical interactions. The plasticity becomes a new facet of 2D materials for deformable or flexible electronics.

## Abstract (web summary)

It is still challenging to discover plastically deformable inorganic semiconductors. Here, the authors report a high-throughput screening of tens of potential 2D van der Waals crystals that can deform plastically accompanied with experimental verification.

## Introduction

Semiconductors, especially inorganic semiconductors, are endowed with rich electrical, optical, magnetic, and thermal properties, serving as key functional materials for various electronics in the era of intelligence. Somewhat disappointingly, inorganic semiconductors are prevailingly brittle under ambient conditions^1,2^, which severely harmers their processibility and deformability in the fields like flexible, deformable, and hetero-shaped electronics^3,4^. It is not until recently that several inorganic semiconductors have been unveiled to show exceptional room-temperature plasticity/ductility, such as Ag_2_S^5^ and its alloys^6–11^, ZnS crystal in darkness^12^, and InSe crystal^13,14^, reshaping our knowledge of the mechanical properties of inorganic semiconductors^15^.

As a special category of inorganic semiconductors, two-dimensional van der Waals (2D vdW) materials are composed of strongly bonded atomic layers held together by weak interlayer vdW forces^16^, which have wide applications in transistors, memories, sensors, batteries, thermal management etc.^17–22^. Although mono- or few-layer vdW materials show large bending flexibility^15,23^, the bulk forms of 2D vdW semiconductors are usually believed to exhibit poor deformability because of their weak interlayer forces. Therefore, the exceptional plasticity found in InSe vdW bulk crystal is quite stirring: it can be largely compressed and morphed into various shapes while holding the integrity^13^. On the one hand, such plasticity leads to the large deformability in the bulk form; on the other hand, the underlying peculiar chemical interactions may also enhance reliability and stability of the mono- or few-layer forms. Considering the thousands of reported 2D vdW materials^24^, the discovery of plasticity in InSe offers high expectation on the existence of abundant plastic/ductile 2D vdW crystals with diverse properties to meet the requirements for various potential applications. However, the traditional trial-and-error method would take a long time and high cost to discover these novel materials. This is one of the key reasons why only few plastic/ductile inorganic materials have been reported so far^15^.

**Fig. 1:**
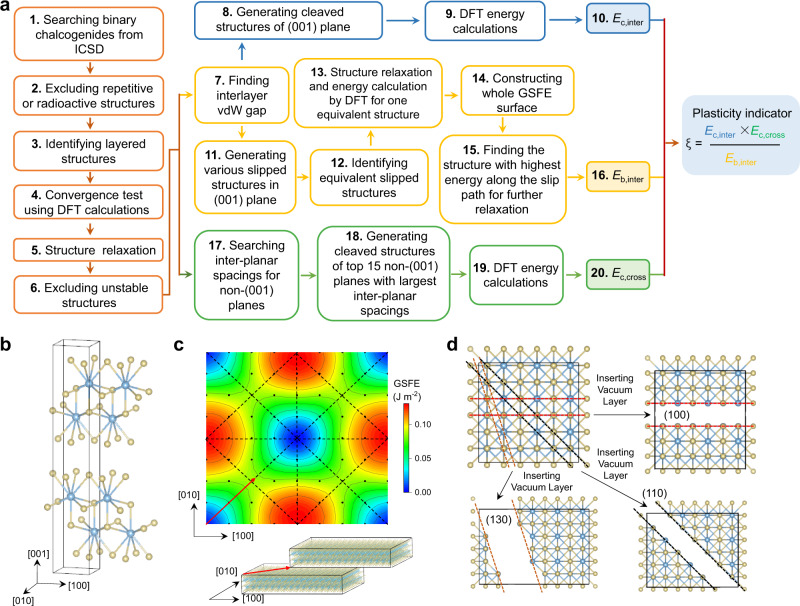
High-throughput methodology to screen 2D vdW chalcogenide crystals with plastic deformability. **a** High-throughput calculation flow. All the steps are automatized except for steps 6 and 15. **b** Crystal structure of CeTe_3_ as a case example. **c** GSFE surface of (001) plane (up) to analyze the interlayer slip (down) for CeTe_3_. The black dots represent slip vectors, corresponding to the various slipped structures. The GSFE surface can be divided into 16 equivalent triangles (see black dash lines) because of the crystal symmetry. **d** Three possible cross-layer cleavage planes for CeTe_3_.

In this work, taking binary semiconducting chalcogenides as the search scope, we develop a high-throughput methodology (Fig. 1) and screen out tens of potential 2D vdW crystals with plastic deformability from the inorganic crystal structure database (ICSD) based on a practical plasticity indicator. The plastic deformability of seven candidates has been verified carefully in experiment and part of them are singled out as case studies to elaborately probe the detailed mechanical properties as well as the mechanisms of plasticity on both macroscopic and microscopic levels.

## Results

### Screening indicator for plastic deformability

**Fig. 2:**
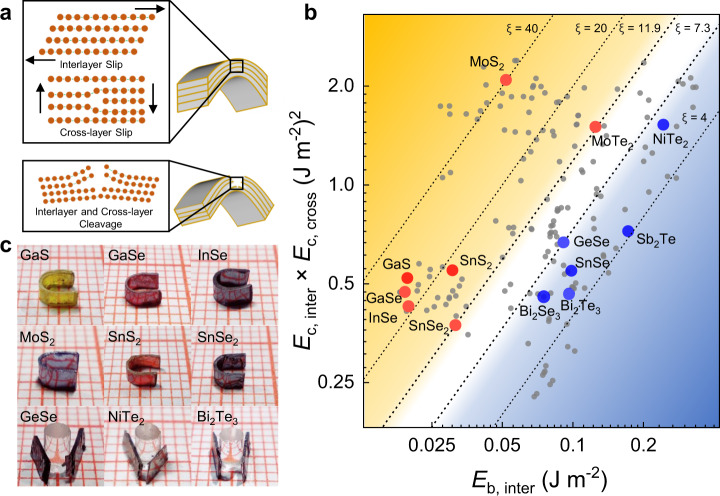
Plastically deformable 2D vdW chalcogenide crystals discovered by high-throughput screening. **a** Schematics of the plastic (up) and brittle (down) bending deformations. The interlayer/cross-layer slips and/or cleavages are highlighted. **b** *E*_c,inter_ × *E*_c,cross_ vs. *E*_b,inter_ for 2D vdW crystals. Each point represents a 2D vdW material with the detailed data shown in Supplementary Table 1. The plastic and brittle crystals verified in experiment are marked by red and blue dots, respectively. **c** Photos of bent plastic and brittle crystals with a bending radius of 1 mm. The smallest grid in the background denotes 1 mm.

High-throughput calculation is an emerging efficient paradigm that can rapidly screen and predict required materials with target properties^25–27^. To quickly evaluate whether a material has room-temperature plasticity or not, a valid and technologically accessible performance indicator is required. The plastic deformation or fracture of 2D vdW crystals is usually induced by the load along *c* axis, exemplified by the bending deformation shown in Fig. 2a. To release the stress, the interlayer and/or cross-layer cleavages tend to happen for brittle crystals, while interlayer and cross-layer slips would take place in plastically deformable crystals. Conceptually, a combination of a large cleavage energy (*E*_c_, defined as the energy per unit area to form two free surfaces when a crystal cleaves) and a low slip barrier energy (*E*_b_, defined as the highest energy per unit area to overcome along the slip path) has been adopted to evaluate the deformability of a certain crystallographic planes in Ag_2_S and InSe, and a deformability factor Ξ = *E*_c_/*E*_b_·(1/*E*_in_) (*E*_in_ is the in-plane modulus) has been consequently proposed to predict potentially deformable semiconductors^5,13^. However, such a factor only considers the slip and cleavage of interlayer planes without including cross-layer slip and cleavage. Furthermore, it is hard to quickly obtain *E*_in_ via high-throughput calculations. As shown in Fig. 2a, a plastically deformable 2D vdW crystal should possess large interlayer (*E*_c,inter_) and cross-layer (*E*_c,cross_) cleavage energies, but small inter-layer (*E*_b,inter_) and cross-layer (*E*_b,cross_) slip barrier energies. A combined index (*E*_c,inter_ × *E*_c,cross_)/(*E*_b,inter_ × *E*_b,cross_) can thus be spontaneously raised to predict 2D vdW materials with plasticity.

In accordance with the conventional realm of material mechanics, the slip and cleavage energies can be quantified by the generalized stacking fault energy (GSFE)^28^ and surface energy (2γ_s_)^29^, respectively. For 2D vdW materials, it is easy to obtain *E*_c,inter_ by inserting vacuum layers, but difficult to compute *E*_b,inter_ because it depends on the slip path in (001) plane. Here, we firstly obtain the GSFE surface of (001) plane using the tilt-cell method^30^; then we identify the minimum energy path (MEP) and the highest energy in this path is taken as *E*_b,inter_^31^. For *E*_c,cross_, the vacuum layers are required to insert into various non-(001) spacings for density functional theory (DFT) calculations to obtain the minimum cleavage energy. For *E*_b,cross_, the GSFE surfaces of various potential non-(001) planes are required to deduce possible MEPs and corresponding barrier energies; then the lowest barrier energy is taken as *E*_b,cross_. Obviously, this procedure is too tough to be realized by high-throughput calculations, especially for complex-structured crystals. Also considering that the interlayer slip is much easier to take place than cross-layer slip (*E*_b,cross_»*E*_b,inter_) in the deformation of 2D vdW crystals, the parameter *E*_b,cross_ can be laid aside for the initial high-throughput calculations without obviously affecting the screening of potentially plastic 2D materials. Therefore, we use the modified index *ξ* = *E*_c,inter_ × *E*_c,cross_/*E*_b,inter_ as a plastic deformability indicator for the high-throughput screening. Although such an index is not an all-embracing criterion, it can give a rough and quick assessment of potentially plastic van der Waals crystals.

### High-throughput screening

The flow of high-throughput methodology is shown in Fig. 1a with the details described in Methods. It includes identifying layered structures of binary chalcogenides, convergence test, structure relaxation, discarding unstable structures (step 1–6), and calculations of *E*_c,inter_ (steps 7–10), *E*_b,inter_ (steps 7, 11–16), and *E*_c,cross_ (steps 17–20). Automated computation programs using the python language under the pymatgen framework^32^ are developed for the whole process except for steps 6 and 15. Furthermore, in order to save computation time and cost, specific automated subroutines for the calculations of *E*_c,inter_, *E*_b,inter_, and *E*_c,cross_ are created. The codes are available as Supplementary Software 1. Here, 2D vdW CeTe_3_, with its crystal structure shown in Fig. 1b, is taken as an example to illustrate the automatic algorithm. The interlayer vdW gap is found by the program firstly. For *E*_c,inter_, an 8 Å thick vacuum layer is inserted to generate the cleaved structure (steps 7 and 8) for further DFT calculations. For *E*_b,inter_, various slipped structures in (001) planes are generated (see Fig. 1c) and then the equivalent slipped structures are recognized by invoking the SPAP program^33^ and then placed in one same group. Finally, only one structure in each group is used for structural relaxation and DFT calculations to construct the whole GSFE surface (see Fig. 1c). This strategy can reduce the computation time and cost by ~70% in average (Supplementary Fig. 1a). For *E*_c,cross_, the interplanar spacings of various non-(001) planes are firstly found by the program. Considering that large interplanar spacing usually leads to small cleavage energy in crystals, we simplify the calculations by picking the top 15 non-(001) planes with largest interplanar spacings to calculate their cleavage energies (steps 17-19, Fig. 1d). The lowest value is thus identified as material’s *E*_c,cross_ in the high-throughput calculations. This approach circumvents the calculations of huge number of possible cleavage planes, and its validity is substantiated by checking the randomly selected 20 2D vdW materials (Supplementary Fig. 1b).

248 layered candidates are identified from 3451 binary chalcogenide structures in the ICSD database. After excluding those containing radioactive elements or with unstable structures, 107 types of materials with 172 ICSD structures in total are left to perform high-throughput calculations. The *E*_c,inter_ × *E*_c,cross_ *vs E*_b,inter_ relation is plotted in Fig. 2b, in which the top-left area (higher *ξ* = *E*_c,inter_ × *E*_c,cross_/*E*_b,inter_) and bottom-right area (lower *ξ*) represent plastic and brittle materials, respectively. To check the validity of this prediction, 13 kinds of single-crystalline materials are obtained in experiment and their deformability is preliminarily evaluated. The materials’ preparation and characterization details are given in Methods and Supplementary Figs. 2, 3. As shown in Fig. 2c, the crystals with the thickness of 0.2–0.4 mm are bent along *c* axis around a cylinder with a diameter of *ϕ* 2 mm. Apart from InSe, another 6 materials, i.e., GaS, GaSe, SnS_2_, SnSe_2_, MoS_2_, and MoTe_2_, can be plastically bent, which are marked by the red dots in Fig. 2b. By contrast, the other materials, such as GeSe, NiTe_2_, and Bi_2_Te_3_, show cross-layer fracture and interlayer cracks, which are marked by blue dots in Fig. 2b. A hazy boundary is highlighted in white between plastic and brittle materials. In the up-left side area with *ξ* ≥ 11.9, the vdW crystals are likely to be plastic for totally 36 materials with 85 ICSD structures. In contrast, on the down-right side with *ξ* ≤ 7.3, the crystals tend to be brittle. The region between the two lines can be regarded as the transitional area and it is difficult to predict whether the materials in this area are plastic or brittle.

### Mechanical properties

**Fig. 3:**
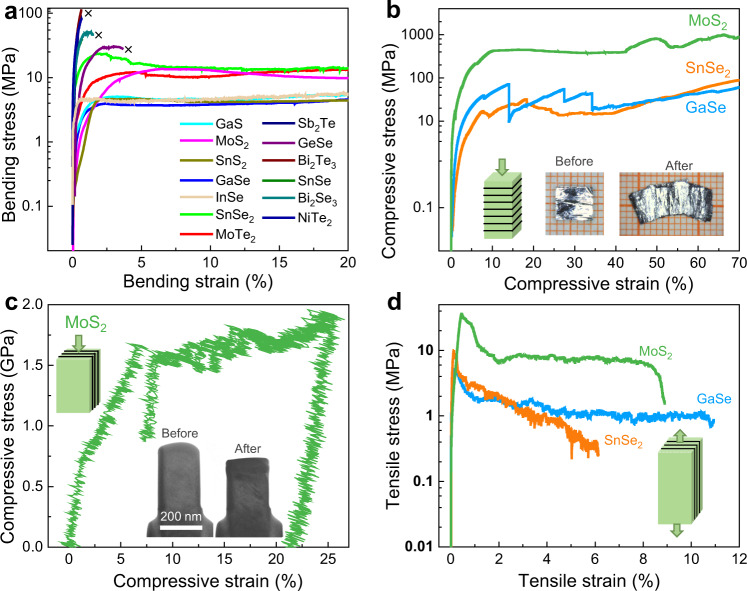
Mechanical properties of selected 2D vdW crystals. **a** Three-point bending stress-strain curves for the selected crystals. **b** Compressive stress-strain curves for MoS_2_, GaSe, and SnSe_2_ crystals. The inset photos show a MoS_2_ crystal before and after compression. **c** In situ compressive stress-strain curve of a MoS_2_ small pillar. The inset shows the specimen transmission electron microscopy (TEM) images before and after test. **d** Tensile stress-strain curves for MoS_2_, GaSe, and SnSe_2_ crystals.

We perform detailed mechanical characterizations on the 13 materials selected above. The test specimens are of non-standard geometries due to their small crystal sizes. For the plastically deformable crystals, the engineering bending strains exceed 20% (see Fig. 3a). In fact, the measured bending strain is restricted by the geometry of apparatus and specimens, which is less than the maximum value before fracture. By contrast, the brittle crystals (e.g., SnSe, Bi_2_Te_3_, NiTe_2_, and Sb_2_Te) are easily fractured upon a small bending strain <5%. As summarized in Supplementary Fig. 1c, the *ξ* index can well distinguish the plastic or brittle vdW crystals. We further single out MoS_2_, GaSe, and SnSe_2_ crystals to carry out uniaxial compressive and tensile tests. As shown in Fig. 3b, the compressibility is noticeable in the three materials with the engineering strain exceeding 70% along *c*-axis, comparable with the InSe crystal^13^. Because of the difficulty in preparing large bulk specimens for in-plane compression tests, small micro-machined MoS_2_ pillars are used (Fig. 3c). It shows a uniform deformation with a uniaxial compressive strain larger than 20%. For tensile tests, bar-shaped crystals are stretched along the in-plane direction (Fig. 3d). The tensile stress first increases to a peak and then rapidly decreases to a plateau, which should be caused by the breaking of a finite number of layers. Then the stress does not drop to zero but keeps roughly unchanged or slightly decreased with the maximum value above 6%, which may be related to the continuous interlayer slip.

### Plastic deformation mechanisms

**Fig. 4:**
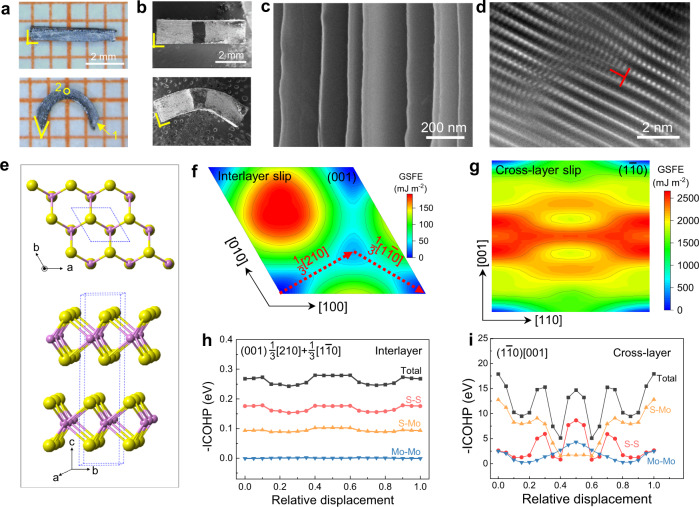
Plasticity mechanism of MoS_2_. **a** Optical images of a bar-shaped MoS_2_ crystal before and after bending. **b** Scanning electron microscopy (SEM) images of an Ag bar before and after bending. Black ink is marked on sample’s top surface to help to fix the position. **c** SEM image of area 1 in **a**. **d**, Inverse Fourier transform of the dark-field scanning transmission electron microscopy (IFT-DF-STEM) image of area 2 in **a**. **e** Crystal structure of 2H-MoS_2_. **f** Generalized stacking fault energy (GSFE) surface of MoS_2_ (001) plane. **g** GSFE surface of MoS_2_ ($1\bar{1}0$) plane. **h** The integrated crystal orbital Hamilton population (-ICOHP) analysis of the interlayer chemical interactions between the slip planes for (001)1/3[210]+1/3[$1\bar{1}0$] slip system shown in **f**. **i** -ICOHP analysis of the chemical interactions between the slip planes for ($1\bar{1}0$)[001] slip system shown in **g**.

The plastic deformation mechanisms of these vdW crystals are studied from both macroscopic and microscopic perspectives. Figure 4a, b show the photographs of rectangle bars before and after bending for MoS_2_ and Ag (a typical metal with superb plasticity), respectively. For Ag, the two ends of the bar-shaped sample are barely affected and a nice 90° angle (marked in yellow) is maintained after bending, suggesting a localized plastic deformation. This is coordinated by the multiple available slip systems in metals^34^. In contrast, for the MoS_2_ after bending, the angles between the end and upper/lower surfaces obviously departure from 90°, suggesting that the plastic deformation extends through the whole sample even at two ends. This phenomenon is mainly caused by material’s interlayer slips, which result in strongly anisotropic plastic deformations. The interlayer slip is further evidenced by the clear glide steps shown in Fig. 4c at end surface. Meanwhile, the cross-layer slip is traced by meticulous microscopic characterizations on a severely strained cross-sectional area (area 2 in Fig. 4a). As shown in Fig. 4d, clear lattice strips are observed and some of them are obviously curved. Notably, a discontinuous layer forms the half-atomic plane for an edge dislocation with the Burgers vector roughly parallel to *c*-axis. The collective motion of these dislocations would render the cross-layer slip^13^. Similar behaviors are also found in GaSe and SnSe_2_ (Supplementary Figs. 4a–c and 5a–c). The above experimental tests are consistent with our model shown in Fig. 2a embracing both interlayer and cross-layer slips for 2D vdW materials.

The *E*_c,inter_, *E*_b,inter_, and *E*_c,cross_ involved in *ξ* indicator are re-calculated using high precision setups for the selected materials (see Methods for details). The data are consistent with the high-throughput results for all the materials except a slightly larger deviation for SnSe_2_ (Supplementary Table 2). Furthermore, the cross-layer slip barrier energy *E*_b,cross_ is calculated for the six selected materials. Several non-(001) planes with low-cleavage energies are picked and their GSFE surfaces, MEPs, and *E*_b,cross_ are calculated (see Methods and Supplementary Table 3). This will update the slip/cleavage planes and relevant energies by high-throughput calculations, since the cross-layer plane with lowest *E*_b,cross_ may not be the one with lowest *E*_c,cross_. After including both the interlayer and the cross-layer slip/cleavage behaviors, the enriched indicator (*E*_c,inter_ × *E*_c,cross_)/(*E*_b,inter_ × *E*_b,cross_) is obtained, which can also well distinguish plastic and brittle vdW crystals. This in turn substantiates the rationality of our model and the validity of the high-throughput screening.

The mechanism of plasticity is further inspected for MoS_2_, GaSe, and SnSe_2_ crystals as case studies. The interlayer slip paths in (001) plane could be 1/3[210]+1/3[$1\bar{1}0$] for MoS_2_ (Fig. 4f), and 1/3[120]+1/3[$\bar{1}10$] for GaSe and SnSe_2_ (Supplementary Figs. 4e and 5e). *E*_c,inter_ is much larger than *E*_b,inter_ (Supplementary Table 2), enabling interlayer slip without fracture for these 2D crystals. For cross-layer slip of MoS_2_, the (105)[$\bar{5}01$] system used in high-throughput screening has the *E*_b,cross_ of 2.87 J m^−2^ and *E*_c,cross_ of 3.80 J m^−2^ (Supplementary Fig. 6 and Table 3). However, ($1\bar{1}0$)[001] slip system also shows a comparable *E*_b,cross_ of 2.94 J m^−2^ but a much large *E*_c,cross_ of 4.42 J m^−2^ (Supplementary Tables 2 & 3). Both of them thus could be possible cross-layer slip systems. For GaSe and SnSe_2_, the possible cross-layer slip systems are likely to be (1$\bar{1}$0)[001] and ($\bar{1}02$)[211], respectively (Supplementary Tables 2 and 3).

The chemical interactions during interlayer and cross-layer slips are further studied by charge density and integrated crystal orbital Hamilton population (-ICOHP) analyses^35^. 2H-MoS_2_ adopts a two-layer hexagonal unit cell stacking along *c*-axis in ABAB… sequence (Fig. 4e)^36^. Each layer is composed by three S-Mo-S atomic planes, where the S atoms form a triangular prism with Mo at the center. During interlayer slip, the total -ICOHP fluctuates to a small extent (Fig. 4h) while maintaining a relatively large value, suggesting a small variation in the total chemical bonding strength. This is consistent with the obtained low *E*_b,inter_ and relatively large *E*_c,inter_. During cross-layer slip, the total -ICOHP of ($1\bar{1}0$)[001] system fluctuates to a certain extent (Fig. 4i), but still possesses a relatively strong chemical bonding strength. These data are consistent with the calculated small *E*_b_ and relatively large *E*_c_ values. Detailed study on chemical interactions shows that the initial Mo-S bonds break during slip, but new Mo-Mo and S-S bonds are formed, leading to the overall relatively strong bonding strength between adjacent planes to hold them together during slip (Fig. 4i and Supplementary Figs. 8a–d). For the (105)[$\bar{5}01$] slip system of MoS_2_ (see Supplementary Fig. 6), the total chemical bonding strength drops largely during slip as compared to the ($1\bar{1}0$)[001] system, indicating that the latter is more feasible for cross-layer slip. Similar results and conclusions are also found in GaSe and SnSe_2_ (Supplementary Figs. 7 and 8). In GaSe, the primary Ga-Se bond breaks while new Ga-Ga and Se-Se bonds form during slip (Supplementary Figs. 7c and 8e–h). In SnSe_2_, the initial Sn1-Se1 and Sn2-Se2 bonds break while Sn3-Se3 and Sn2-Sn4 bonds form during slip (Supplementary Figs. 7d and 8i–l).

## Discussion

2D vdW crystals possess some common plastic deformation behaviors and mechanisms. It combines the interlayer and cross-layer slips. The former is much easier to be activated than the latter, leading to a strong anisotropic deformation as compared with metals. The cross-layer atomic interactions play a key role when maintaining material’s integrality during plastic deformation. Such cross-layer chemical bonding also provides a considerable interaction strength for material’s reliability and stability in both the bulk and few-layer forms.

The high-throughput methodology and the deformability factor developed in this work can be also used to explore more plastically deformable materials beyond these 2D vdW crystals through calculating the slip and cleavage energies of multiple possible slip systems. In terms of the calculation accuracy and speed, how to quickly and efficiently calculate the slip barrier energies (*E*_b,cross_) of multiple possible slip systems is very challenging. Furthermore, the plasticity is greatly affected by material’s defects such as dislocations, which should be incorporated into the high-throughput methodology in the future.

In summary, we have reported tens of potential 2D vdW semiconducting chalcogenides with plastic deformability via a high-throughput screening strategy. This study indicates that plasticity is becoming a new facet of 2D vdW crystals alongside the rich electrical, optical, thermal, and magnetic properties, which will speed up their applications in deformable, flexible, or hetero-shaped electronics. The physical mechanisms behind the plasticity can also help understand and design various electronic devices with good reliability and stability based on these advanced 2D vdW materials. Moreover, the screening methodology can be also extended to explore non-2D materials with plastic deformability.

## Methods

### High-throughput computations

The flow of high-throughput calculation methodology includes searching and retrieving binary chalcogenide structures from the Inorganic Crystal Structure Database (ICSD), excluding repetitive structures and structures containing radioactive elements (Z > 83), convergence test, structure relaxation, discarding unstable structures and the calculations of *E*_c,inter_, *E*_c,cross_, and *E*_b,inter_. The details are descried below. All steps except step 6 and 15 are automatically performed using the python program language under pymatgen framework^32^. All DFT calculations are carried out using the Vienna ab initio simulation package (VASP)^37^ with the projector augmented wave method^38^. The PBE functional is employed using the DFT-D3BJ van der Waals correction^39^.

3451 binary chalcogenide crystal structures at ambient conditions are searched and retrieved from ICSD (step 1). 1297 unique structures are firstly found by the structure prototype analysis package (SPAP)^33^ and used thereafter. The structures containing elements with atomic numbers larger than 83 are excluded since they are radioactive (step 2). Layered structures are identified from the remaining structures by the approach developed by Mounet^24^ and Cheon^40^. Finally, 248 layered structures are identified and used in the next step (step 3). For the convergence test (step 4), the k-point density is gradually increased to ensure that the energy convergences of 1, 5, and 10 meV atom^−1^ are realized for 86.6%, 99.4%, and 100% of the materials, respectively. Then the structures are fully relaxed using a criterion of 0.01 eV Å^−1^ and 51 unstable structures are discarded (steps 5 and 6).

To calculate *E*_c,inter_, the interlayer van der Waals gap is firstly located by the approach developed by Mounet^24^ and Cheon^40^. Then a vacuum layer with a thickness of 8 Å is automatically inserted to generate the cleaved structure (steps 7 and 8). The energies of initial structure (*E*_initial_) and cleaved structure (*E*_cleaved_) are obtained by DFT calculations. *E*_c,inter_ is calculated by (*E*_initial_ - *E*_cleaved_)/*A*, in which *A* is the cleavage surface area (steps 9 and 10).

To obtain interlayer slip barrier energy *E*_b,inter_, the GSFE (generalized stacking fault energy) surface is required to find the easiest slip pathway, known as the minimum energy path (MEP). An automated sub-program is developed. At first, various slipped structures are generated by the tilt-cell method^30^ (step 11). Then, the equivalent slipped structures are recognized by invoking the SPAP program^33^ and placed in one same group (step 12). Only one structure in each group is selected for structural relaxation using a force criterion of 0.03 eV Å^−1^ and DFT energy calculations (step 13). Finally, the data are used for other equivalent structures to construct the whole GSFE surface automatically (step 14). This strategy can decrease the calculation time and cost by ~70% in average (Supplementary Fig. 1b). The structures with the highest energy along the MEPs are found manually and then further relaxed to a target with a force criterion of 0.01 eV Å^−1^ to obtain accurate slip energy barriers (steps 15 and 16).

To calculate *E*_c,cross_, an automated program is developed to search non-(001) inter-planar spacings and generate cleaved structures by inserting an 8 Å-thick vacuum layer (step 17). The cleavage energies of these cleaved structures are obtained by DFT calculations and the lowest one, min(*E*_c_), is taken as *E*_c,cross_ (steps 18 and 19). The amount of non-(001) planes with various interplanar spacings in material’s crystal structure is huge. Therefore, it is rather time-consuming if all of them are included for DFT calculations. Here, we find that the crystallographic planes with large interplanar spacing usually tend to low cleavage energies. Then we randomly select 20 2D vdW materials to test the cleavage energies of non-(001) planes with different interplanar spacings by DFT calculations. The min(*E*_c_) appears within the planes with top 14 largest interplanar spacings for all the materials (Supplementary Fig. 1b). Therefore, top 15 planes with the largest interplanar spacings are used in the high-throughput calculations to obtain *E*_c,cross_. This strategy can efficiently and reliably obtain *E*_c,cross_ for hundreds of materials. 17 structures are excluded since they have more than 500 atoms in their cleaved structures, which are too complicated for DFT calculations.

### Refined calculations and charge analysis

Refined calculations of *E*_c,inter_, *E*_c,cross_, and *E*_b,inter_ are carried out for 13 selected materials. The cut-off energies and converged k-points density are increased towards a converge value of 0.1 meV atom^−1^ as compared with the high-throughput calculations (1 meV atom^−1^). The *E*_b,inter_ is calculated by using the slab method^41^ to generate the slipped structures. The data by the precise method and high-throughput method are listed in Supplementary Table 2. The slip barrier energies *E*_b,cross_ of possible cross-layer slip systems are also calculated for the selected 6 materials using the slab method with the data given in Supplementary Table 2. The charge density diagrams are generated by performing self-consistent calculations on the relaxed structures. The crystal orbital Hamilton population (COHP) analysis is performed using the LOBSTER program^42^. The basis set for COHP analysis is VASPFit2015 and the charge spilling is smaller than 2%.

The cross-sectional charge density is plotted in Supplementary Fig. 8 to analyze the chemical interactions during cross-layer slips for MoS_2_, GaSe, and SnSe_2_. For the MoS_2_ ($1\bar{1}0$)[001] slip system, the slip plane ($1\bar{1}0$) is perpendicular to page (the (110) plane), which is marked by the dashed red line. The strong Mo-S bonds (Mo1-S1 and Mo1-S2) correspond to a highest -ICOHP (Fig. 4i) at initial state. When slipping, the left and right parts move oppositely along [001] direction (*x* increasing; see the red arrows in Supplementary Fig. 8a). During this process, Mo1-S2 and Mo1-S1 bonds generally stretch and almost break till *x* = 0.2 and 0.4, respectively, together leading to decreased Mo-S bond strength in total (Fig. 4i). By contrast, S3 and S4 atoms start to interact with S1 and S2 atoms, respectively, forming new, strong S-S bonding at *x* = 0.5 (Fig. 4i and Supplementary Fig. 8d). Similarly, Mo1 at lower layer also interacts with Mo2 at upper layer to form a new Mo-Mo bond at *x* = 0.5.

### Crystal growth and material preparation

InSe single crystal is grown by a modified Bridgeman methods^13^. For GaS, GaSe, SnSe_2_, SnSe, SnS_2_, Bi_2_Se_3_, Bi_2_Te_3_, Sb_2_Te, and NiTe_2_, the single crystals are grown via the following procedures. Raw materials in stoichiometric ratio are loaded in a silica glass tube and sealed in vacuum. The tubes are placed in a vertical furnace with a temperature gradient (~10 °C cm^−1^) and then heated to the temperature 50 °C above its melting point with a heating rate <30 °C h^−1^. The melts are kept at this temperature for 5 h and then slowly cooled to the temperature 50 °C below its melting point. After that, the tubes are naturally cooled in furnace except that NiTe_2_ is quenched into the water.

GeSe single crystal is grown by the physical vapor transport method. Stoichiometric Ge and Se are loaded into a silica glass tube and sealed under vacuum. The tube is put into a horizontal two-zone furnace with the raw materials at hot end. The furnace is firstly heated to 690 °C for both ends and dwelled at this temperature for 20 h. Subsequently, the temperature of cold end is decreased to 650 °C while keeping hot end at 690 °C. After 40 h, the tube is quenched into the water.

MoS_2_ crystal with shinny cleavage surface is mechanically cleaved and exfoliated from molybdenite minerals. Flux grown MoTe_2_ single crystal is purchased from Shanghai Onway Technology Co., Ltd.

### Structure and composition characterization

The X-ray diffraction patterns are collected on both the cleaved surfaces and powders of vdW crystals by using the Cu Kα source diffractometer (D8 Advance, Bruker®, Germany). Samples’ morphology is examined by the scanning electron microscope (SEM, Verio G4 UC, Thermo Fisher Scientific®, U.S). The chemical composition is checked by energy dispersive spectroscopy (EDS, Oxford Instruments, U.K.) on the cleaved surfaces. The microstructures are further examined by the probe-corrected scanning/transmission electron microscope (S/TEM, HF5000, Hitachi® High-Tech, Japan). The TEM samples are cut and thinned by focused ion beam (Versa 3 DualBeam, FEI®, U.S.).

The XRD patterns and EDS mappings (see Supplementary Figs. 2, 3) show that all the 13 vdW crystals are phase pure with homogenous elemental distributions and decent crystallinity. The actual chemical compositions detected by EDS point analyses are close to the nominal compositions.

### Macroscopic mechanical tests

Three-point bending tests are performed on bar-shaped specimens by using a dynamic mechanical analyzer (Discovery DMA 850, TA Instruments®, U.S.) with a supporting span of 5 mm and a loading rate of 0.1 mm min^−1^. The tensile and compressive tests are performed using a universal testing machine (AGS-X, Shimadzu®, Japan) with a loading rate of 0.1 mm min^−1^. The specimens are of non-standard geometries due to the small crystal size.

### In situ TEM compression tests

The MoS_2_ single crystalline micropillars are fabricated from a pre-thinned bulk single crystal with the loading direction perpendicular to *c* axis. Micromaching is performed by a dual-beam focused ion beam (FIB, Helios Nanolab^TM^ 600, FEI®, U.S.) under 30 keV Ga^+^ ion beam current sequentially decreasing from ~10 nA (coarse cutting) to ~10 pA (fine polishing). The subsequent uniaxial compression test on MoS_2_ single crystalline micropillars is performed by a Brooker-Hysitron PicoIndenter® (PI 95) with a diamond punch inside the FIB chamber inside TEM (JEOL 2100 F, 200 kV). The compression tests are conducted quasi-statically with a constant strain rate of ~5 × 10^−3^ s^−1^. The engineering stress is calculated by dividing the load by the cross-sectional area before deformation, and the engineering strain is defined as the ratio of the deformation displacement of pillar (i.e., the displacement reading minus the contribution from the substrate) to its initial height (the distance from top to the substrate).

## Supplementary information

Supplementary Information

Description of Additional Supplementary Files

Supplementary Software 1

## Data Availability

The data generated in this study are provided in the Supplementary Information.

## Code availability

The computation code is available as Supplementary Software 1.
