# Supplementary material for: High-throughput screening of 2D van der Waals crystals with plastic deformability
Source: Nat Commun. 2022 Dec 5;13:7491. doi: 10.1038/s41467-022-35229-x (PMC9723169; doi:10.1038/s41467-022-35229-x)
Supplement: Supplementary file 1 — Supplementary Information [file 41467_2022_35229_MOESM1_ESM.pdf]

High-throughput screening of 2D van der Waals crystals with plastic deformability

(Supplementary Information)

Zhiqiang Gao[1,2], Tian-Ran Wei[3*], Tingting Deng[1,4], Pengfei Qiu[1,4,5], Wei Xu[6], Yuecun Wang[6], Lidong Chen[1,2,5], Xun Shi[1,3,5*]

[1]State Key Laboratory of High Performance Ceramics and Superfine Microstructure, Shanghai Institute of Ceramics, Chinese Academy of Sciences; Shanghai 200050, China

[2]School of Physical Science and Technology, ShanghaiTech University; Shanghai 201210, China

[3]State Key Laboratory of Metal Matrix Composites, School of Materials Science and Engineering, Shanghai Jiao Tong University; Shanghai 200240, China

[4]School of Chemistry and Materials Science, Hangzhou Institute for Advanced Study, University of Chinese Academy of Sciences; Hangzhou 310024, China

[5]Center of Materials Science and Optoelectronics Engineering, University of Chinese Academy of Sciences; Beijing 100049, China

[6]Center for Advancing Materials Performance from the Nanoscale (CAMP-Nano) & Hysitron Applied Research Center in China (HARCC), State Key Laboratory for Mechanical Behavior of Materials, Xi'an Jiaotong University; Xi'an 710049, China

Email: tianran_wei@sjtu.edu.cn (T.-R.W.); xshi@mail.sic.ac.cn (X.S.)

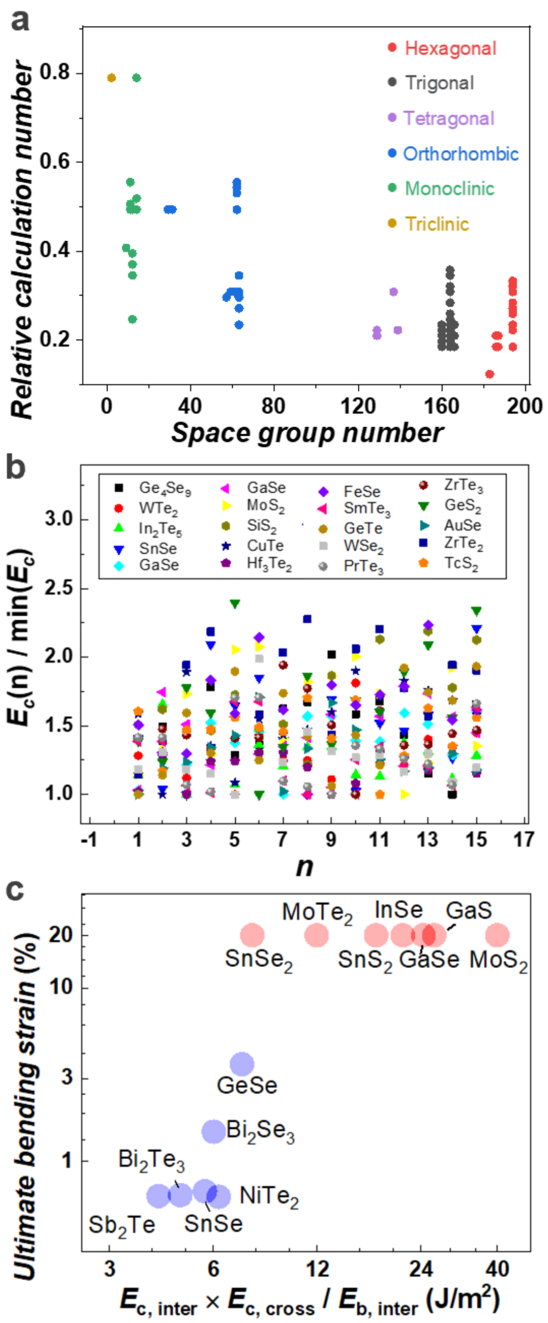

Supplementary Figure 1.

Calculation details of high-throughput screening and plasticity index ζ for various 2D vdW materials. a, Relative calculation numbers for the crystal structures with different space group numbers. The relative calculation number is the ratio of the calculation numbers using the equivalent structure strategy to those by the approach without using equivalent structure strategy. **b,** Normalized cleavage energy $E_c(n)/\min(E_c)$ *vs.* n for the 20 randomly picked 2D vdW materials. Here $\min(E_c) = \min(E_c(1^{st}), E_c(2^{nd}), \ldots, E_c(15^{th}))$, and n is the number of atomic planes in the descent order of interplane spacing. For all the randomly selected 20 materials, $\min(E_c)$ or $E_c(n)/\min(E_c) = 1$ is found when $n \leq 14$. **c,** Experimental bending strain *vs.* the plasticity index ($\zeta = E_{c,inter} E_{c,cross}/E_{b,inter}$) for plastic (red dots) and brittle (blue dots) vdW crystals.

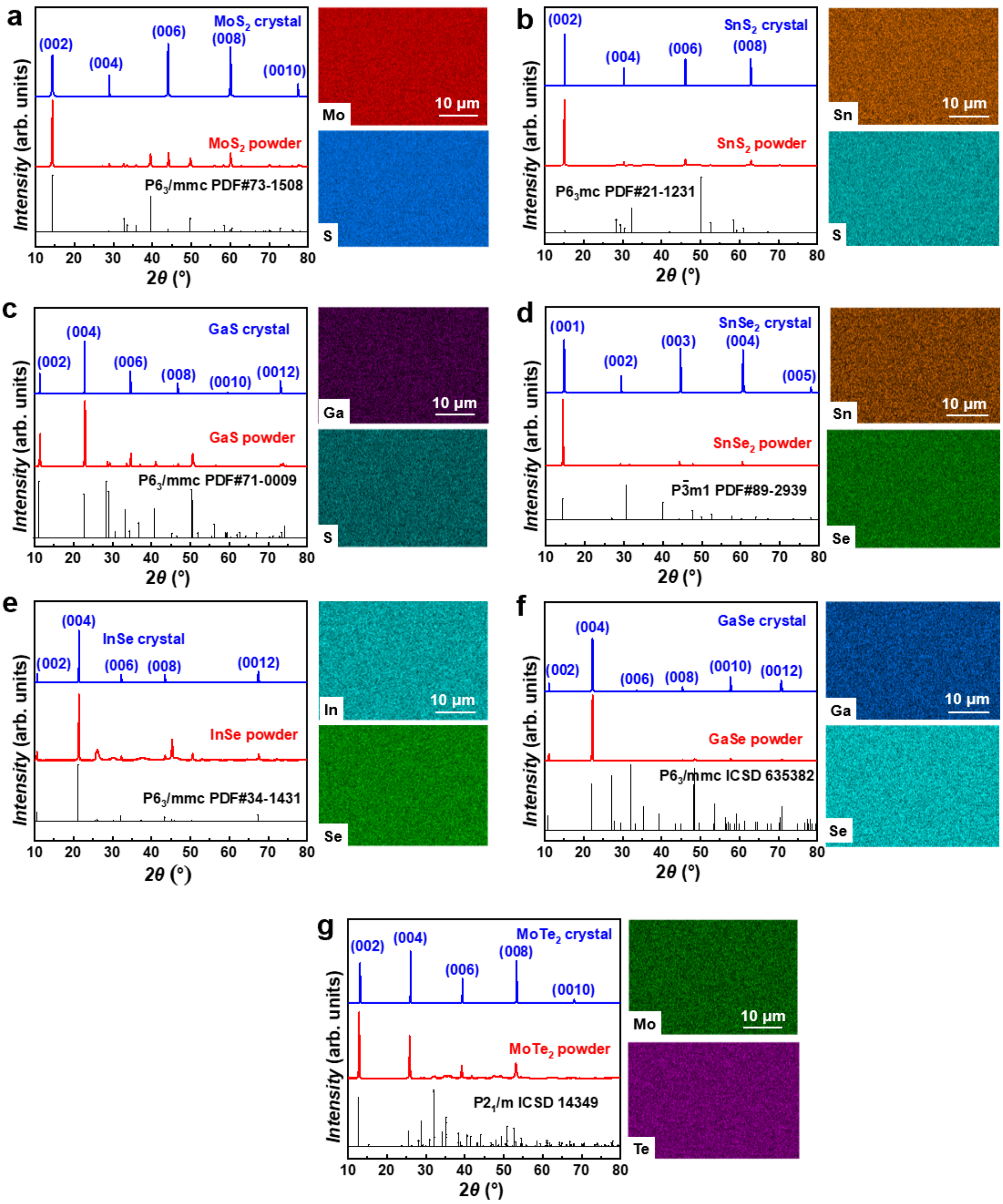

Supplementary Figure 2.

XRD patterns and EDS elemental mappings of plastically deformable vdW crystals. a, MoS_2, **b,** SnS_2, **c,** GaS, **d,** $SnSe_2$, **e,** InSe, **f,** GaSe, and **g,** $MoTe_2$.

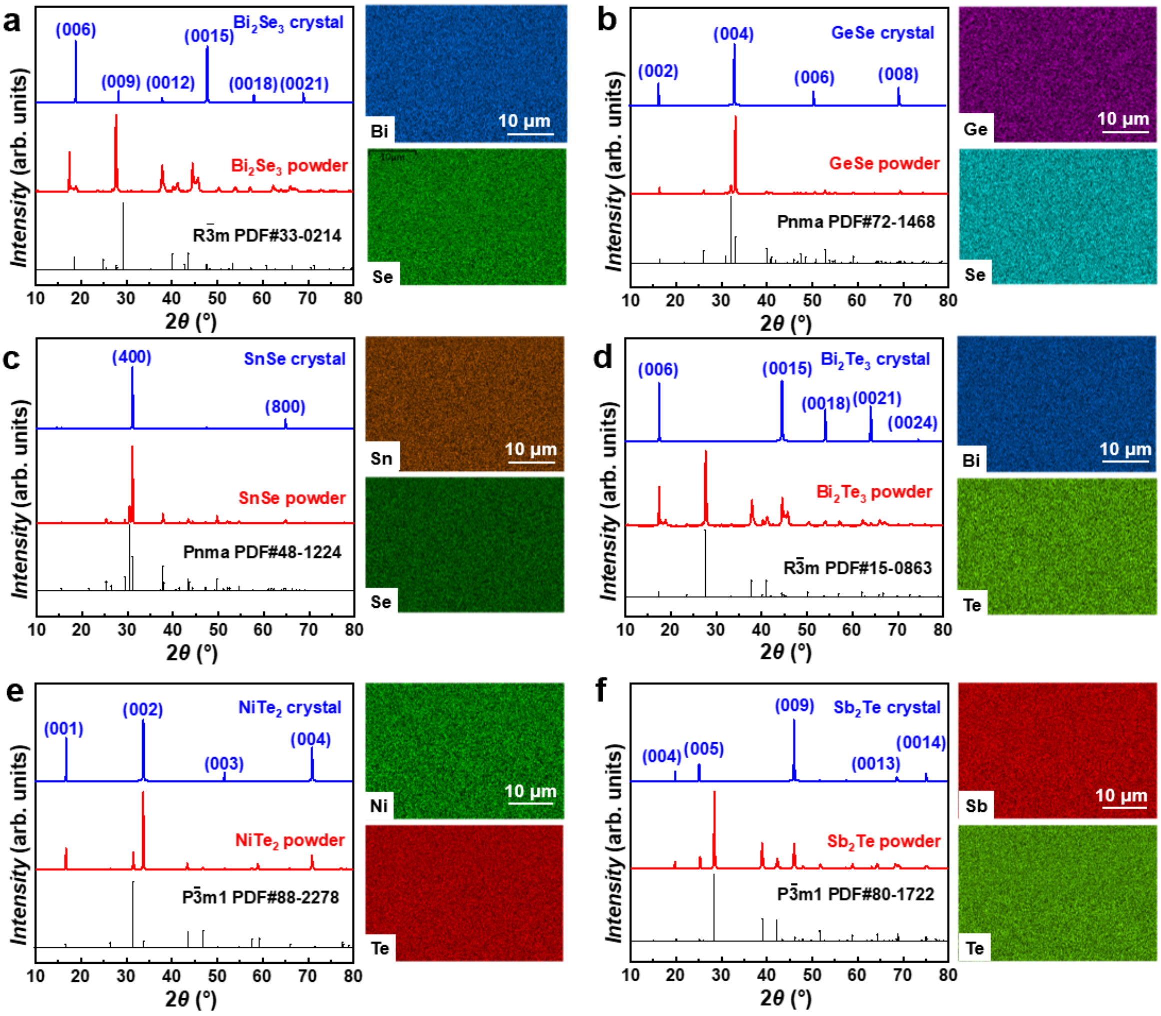

Supplementary Figure 3.

XRD patterns and EDS elemental mapping of brittle vdW crystals. a, Bi_2Se_3, **b,** GeSe, **c,** SnSe, **d,** Bi_2Te_3, **e,** $NiTe_2$ and **f,** Sb_2Te.

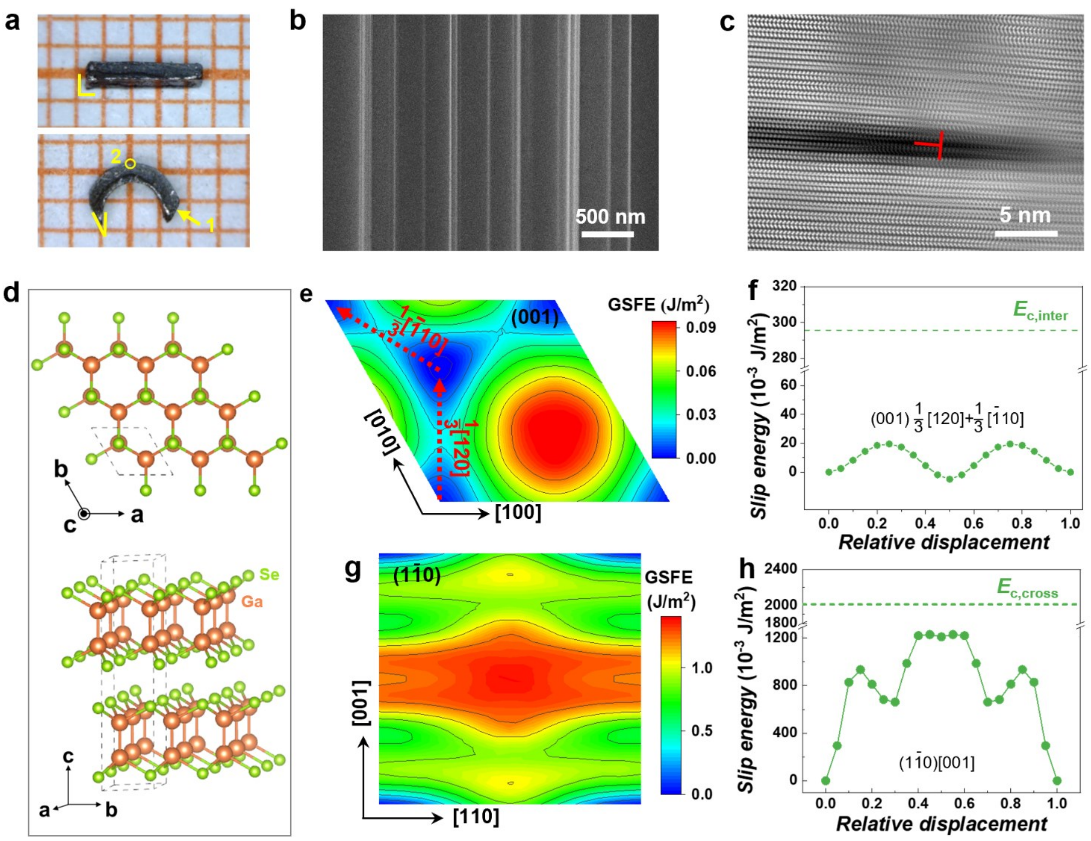

Supplementary Figure 4.

Plastic deformation of GaSe. a, Optical images of a bar-shaped GaSe crystal before and after bending **b,** SEM image of area 1 in **a**. **c,** Inverse Fourier transform of the dark-field scanning transmission electron microscopy (IFT-DF-STEM) image of area 2 in **a**. **d,** Crystal structure of GaSe. **e,** Generalized stacking fault energy (GSFE) surface of GaSe (001) plane. **f,** GSFE of the $(001)1/3[120]+1/3[1\bar{1}0]$ slip path. **g,** GSFE surface of GaSe $(1\bar{1}0)$ plane. **h,** GSFE of GaSe along the $(1\bar{1}0)[001]$ slip path.

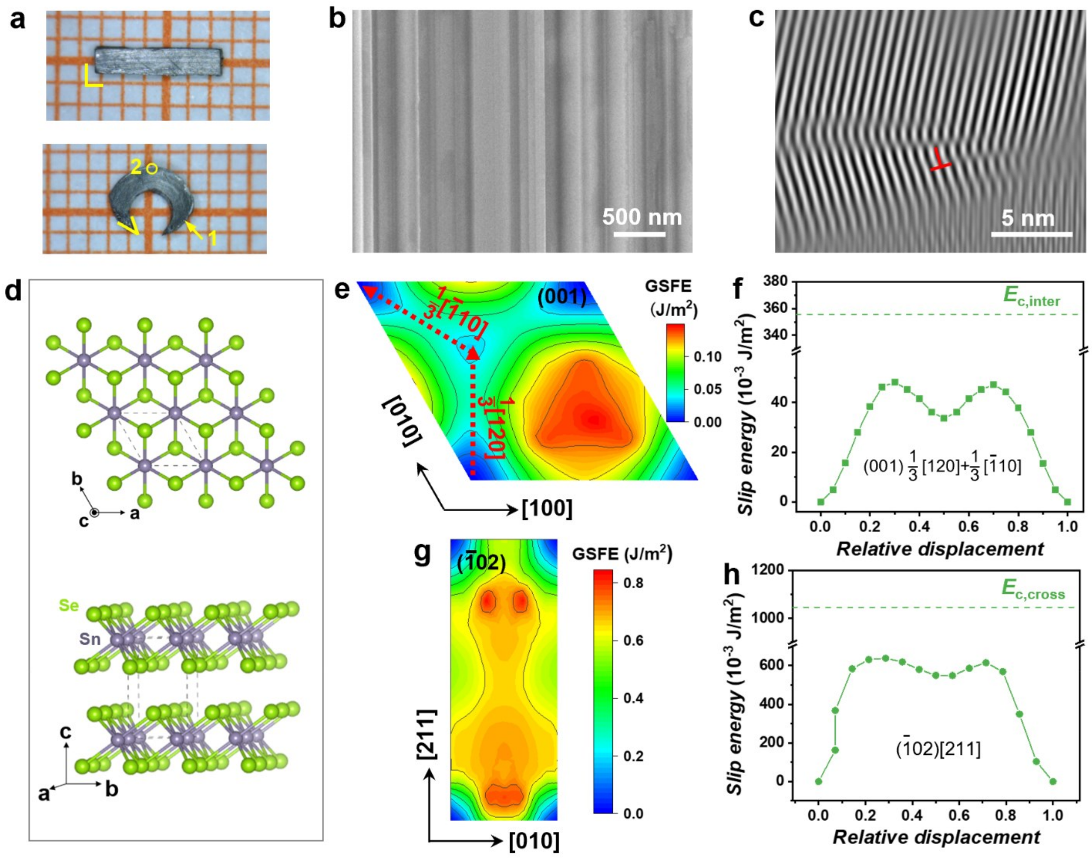

Supplementary Figure 5.

Plastic deformation of $SnSe_2$. a, Optical images of a bar-shaped $SnSe_2$ crystal before and after bending **b,** SEM image of area 1 in **a**. **c,** Inverse Fourier transform of the dark-field scanning transmission electron microscopy (IFT-DF-STEM) image of area 2 in **a**. **d,** Crystal structure of $SnSe_2$. **e,** Generalized stacking fault energy (GSFE) surface of $SnSe_2$ (001) plane. **f,** GSFE of $SnSe_2$ along the $(001)1/3[120]+1/3[\bar{1}10]$ slip path. **g,** GSFE surface of $SnSe_2$ $(\bar{1}02)$ plane. **h,** GSFE of $SnSe_2$ along the $(\bar{1}02)[211]$ slip path.

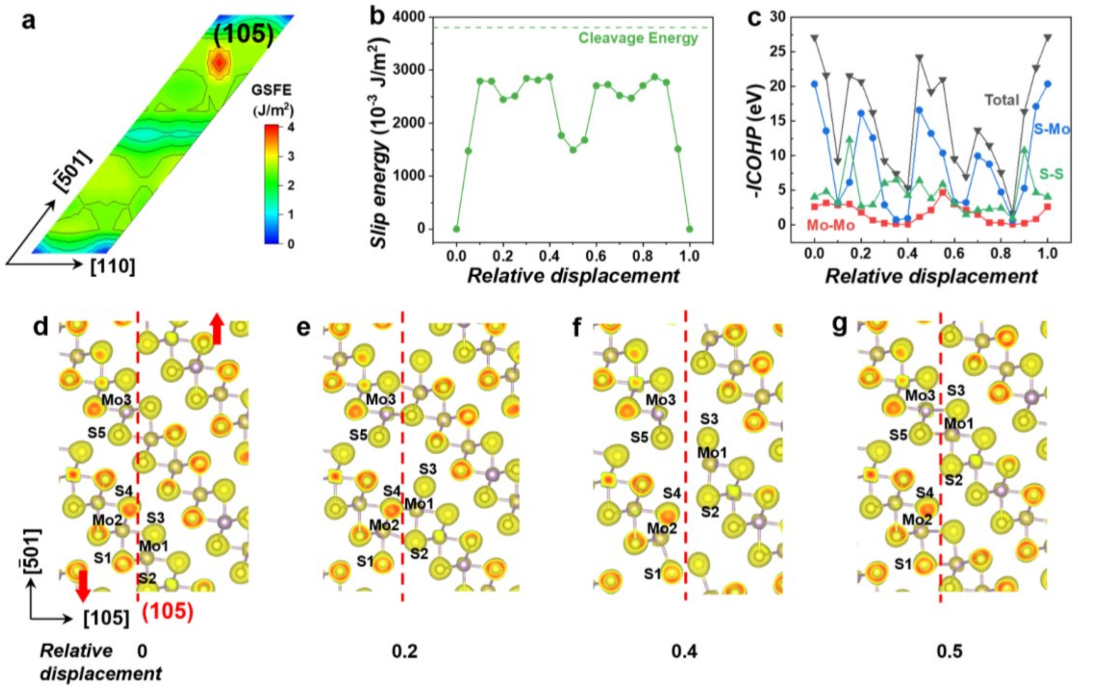

Supplementary Figure 6.

Cross-layer slip of (105)[5̄01] system for MoS_2. a, GSFE surface of MoS_2 (105) plane. **b**, GSFE along the (105)[5̄01] slip path. **c,** The integrated crystal orbital Hamilton population (-ICOHP) analysis of the cross-layer chemical interactions between the slips plane for (105) [5̄01] slip system. **d-g**, Charge density maps for atomic bonding between the slip planes at different relative displacement for (105) [5̄01] slip system. The charge density iso-surface level is 0.8 eÅ^{-3}.

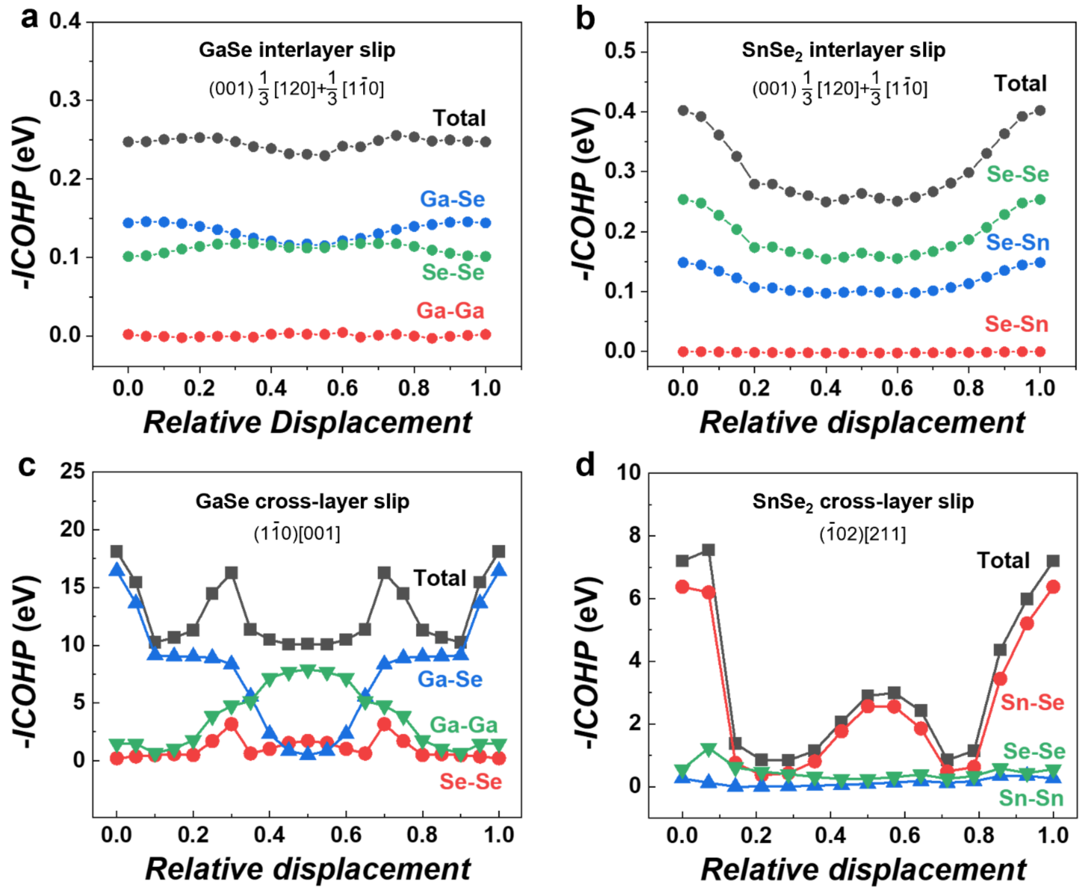

Supplementary Figure 7.

-ICOHP analysis of interlayer and cross-layer chemical interactions between slip planes for GaSe and SnSe. a, GaSe with the slip system of (001) 1/3[120]+1/3[$\bar{1}$10] and **b,** $SnSe_2$ with the slip system of (001) 1/3[120]+1/3[$\bar{1}$10]. **c**, GaSe with the slip system of (1$\bar{1}$0)[001] and **d,** $SnSe_2$ with the slip system of ($\bar{1}$02)[211].

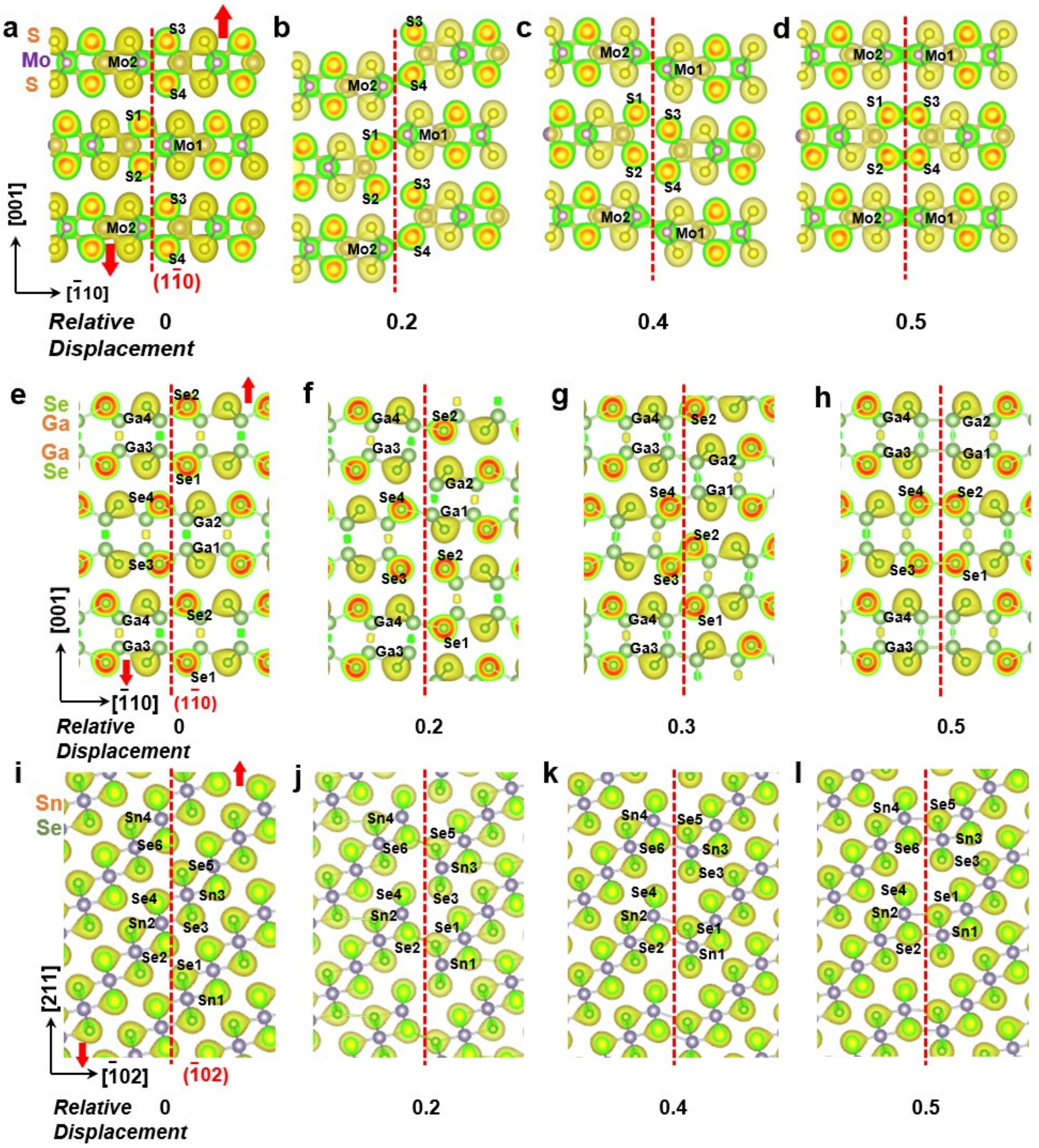

Supplementary Figure 8.

Charge analysis for cross-layer slip in MoS_2, GaSe and $SnSe_2$ crystals. Charge density maps for atomic bonding between the slip planes at different relative displacement for (**a-d**) $(1\bar{1}0)[001]$ slip system in MoS_2, (**e-h**) $(1\bar{1}0)[001]$ slip system in GaSe, and (**i-l**) $(\bar{1}02)[211]$ slip system in $SnSe_2$. The charge density iso-surface levels are 0.45 $e\text{Å}^{-3}$ for (**a-d**), 0.34 $e\text{Å}^{-3}$ for (**e-h**) and 0.8 $e\text{Å}^{-3}$ for (**i-l**).

Supplementary Table 1.

Materials, ICSD ID, space group, $E_{c,cross}$, $E_{c,inter}$, $E_{b,inter}$, and ξ index calculated by high-throughput methods for binary vdW chalcogenides.

Material	ICSD ID	Space group	$E_{c,cross}$ (J m^{-2})	$E_{c,inter}$ (J m^{-2})	$E_{b,inter}$ (J m^{-2})	ξ (J m^{-2})	Plastic or Brittle
NbS_2	645321	R3m	2.86	0.61	2.72×10^{-2}	63.75	Plastic
TaS_2	651083	R3m	3.10	0.56	2.98×10^{-2}	58.40	Plastic
WS_2	202367	R3m	3.99	0.57	4.16×10^{-2}	55.06	Plastic
$NbSe_2$	645391	R3m	2.56	0.64	2.99×10^{-2}	55.04	Plastic
MoS_2	43560	R3m	3.69	0.55	4.26×10^{-2}	47.34	Plastic
$NbSe_2$	18131	R3m	2.67	0.62	3.51×10^{-2}	46.85	Plastic
WS_2	56014	$P6_3/mmc$	4.11	0.58	5.66×10^{-2}	42.29	Plastic
$TaSe_2$	659988	R3m	2.74	0.61	4.04×10^{-2}	41.64	Plastic
WS_2	196993	$P6_3/mmc$	4.11	0.58	5.75×10^{-2}	41.56	Plastic
$MoSe_2$	16948	R3m	3.14	0.57	4.29×10^{-2}	41.39	Plastic
$TaSe_2$	24316	$P\bar{6}m2$	2.78	0.57	3.92×10^{-2}	40.45	Plastic
MoS_2	100042	$P6_3/mmc$	3.80	0.55	5.19×10^{-2}	40.27	Plastic
MoS_2	84180	$P6_3/mmc$	3.81	0.55	5.22×10^{-2}	39.97	Plastic
$TaSe_2$	24314	$P6_3/mmc$	3.08	0.62	5.00×10^{-2}	38.07	Plastic
NbS_3	263457	$P2_1/c$	2.44	0.43	2.78×10^{-2}	37.85	Plastic
BS	230149	$R\bar{3}m$	3.33	0.33	3.00×10^{-2}	36.29	Plastic
$TaSe_2$	18130	$P6_3/mmc$	2.82	0.62	4.90×10^{-2}	35.47	Plastic
TaS_2	651101	$P6_3/mmc$	3.22	0.58	5.48×10^{-2}	33.88	Plastic
WSe_2	84182	$P6_3/mmc$	3.50	0.60	6.96×10^{-2}	30.33	Plastic
$MoSe_2$	601045	$P6_3/mmc$	3.24	0.57	6.16×10^{-2}	30.16	Plastic
$TaSe_2$	89367	$P6_3/mmc$	3.10	0.65	6.79×10^{-2}	29.53	Plastic
$TaSe_2$	24317	$P6_3/mmc$	3.10	0.64	6.74×10^{-2}	29.44	Plastic
MoS_2	254956	$P\bar{3}m1$	2.35	0.63	5.11×10^{-2}	28.74	Plastic
TaS_2	52117	R3m	2.85	0.64	6.68×10^{-2}	27.51	Plastic
NbS_2	250595	$P6_3/mmc$	3.27	0.61	7.31×10^{-2}	27.26	Plastic

GaS	173940	$P6_3/mmc$	1.80	0.29	1.97×10^{-2}	26.43	Plastic
$AsSe_2$	428192	R3m	1.14	0.51	2.21×10^{-2}	26.08	Plastic
GaSe	635382	$P6_3/mmc$	1.59	0.30	1.83×10^{-2}	25.76	Plastic
GaSe	41978	$P6_3/mmc$	1.60	0.30	1.84×10^{-2}	25.64	Plastic
$TaSe_2$	24318	R3m	2.51	0.66	6.50×10^{-2}	25.59	Plastic
TaS_2	85323	$P\bar{3}m1$	2.42	0.60	5.67×10^{-2}	25.55	Plastic
NbS_2	43697	$P6_3/mmc$	2.99	0.64	7.60×10^{-2}	25.11	Plastic
GaSe	2002	$P6_3mc$	1.59	0.30	1.92×10^{-2}	24.59	Plastic
GaSe	73388	R3m	1.64	0.30	2.19×10^{-2}	22.64	Plastic
GaS	40824	$R\bar{3}m$	1.88	0.29	2.46×10^{-2}	22.42	Plastic
TaS_2	24757	$R\bar{3}m$	2.39	0.60	6.46×10^{-2}	22.31	Plastic
GaSe	635363	$P\bar{6}m2$	1.60	0.30	2.17×10^{-2}	21.93	Plastic
Ta_2Se	65739	P4/nmm	4.69	0.97	2.11×10^{-1}	21.66	Plastic
TiS_3	42072	$P2_1/m$	1.10	0.48	2.45×10^{-2}	21.47	Plastic
InSe	640499	$P6_3/mmc$	1.44	0.30	1.99×10^{-2}	21.42	Plastic
InSe	185172	$P6_3/mmc$	1.39	0.30	1.95×10^{-2}	21.12	Plastic
$NbSe_2$	16304	$P6_3/mmc$	2.66	0.67	8.54×10^{-2}	20.89	Plastic
$NbSe_2$	645370	$P6_3/mmc$	2.66	0.67	8.73×10^{-2}	20.49	Plastic
NbS_3	2380	$P\bar{1}$	1.30	0.43	2.78×10^{-2}	20.22	Plastic
VS_2	651361	$P\bar{3}m1$	2.20	0.67	7.45×10^{-2}	19.67	Plastic
$TaSe_2$	199414	$P\bar{3}m1$	2.22	0.62	6.99×10^{-2}	19.65	Plastic
$NbSe_2$	18132	$P\bar{6}m2$	3.02	0.62	9.47×10^{-2}	19.61	Plastic
ReS_2	75459	$P\bar{1}$	2.42	0.50	6.16×10^{-2}	19.47	Plastic
$ReSe_2$	66658	$P\bar{1}$	2.48	0.51	6.62×10^{-2}	19.07	Plastic
SnS_2	43003	$P6_3mc$	1.72	0.32	3.06×10^{-2}	17.94	Plastic
MoS_2	26622	$R\bar{3}m$	2.29	0.64	8.21×10^{-2}	17.74	Plastic
WTe_2	653170	$P6_3/mmc$	3.07	0.64	1.12×10^{-1}	17.64	Plastic
GaTe	43328	$P6_3/mmc$	1.36	0.30	2.38×10^{-2}	17.41	Plastic
In_2Se_3	17008	R3m	0.96	1.07	6.00×10^{-2}	17.17	Plastic
InSe	640479	R3m	1.46	0.31	2.65×10^{-2}	17.11	Plastic

NbS_2	250594	$P\bar{3}m1$	2.24	0.63	8.31×10^{-2}	16.99	Plastic
InSe	640503	$P\bar{6}m2$	1.41	0.30	2.56×10^{-2}	16.60	Plastic
HfS_2	603757	$P\bar{3}m1$	1.86	0.42	4.69×10^{-2}	16.56	Plastic
$ZrSe_3$	652233	$P2_1/m$	1.05	0.45	3.07×10^{-2}	15.48	Plastic
$MoTe_2$	25419	$P6_3/mmc$	2.62	0.62	1.05×10^{-1}	15.47	Plastic
$MoTe_2$	15431	$P6_3/mmc$	2.62	0.62	1.06×10^{-1}	15.43	Plastic
TiS_3	604398	$P2_1/m$	1.09	0.47	3.37×10^{-2}	15.33	Plastic
ZrS_3	604573	$P2_1/m$	1.11	0.41	2.97×10^{-2}	15.28	Plastic
$ZrSe_3$	652229	$P2_1/m$	0.99	0.45	2.97×10^{-2}	15.20	Plastic
ZrS_3	42073	$P2_1/m$	1.06	0.41	2.88×10^{-2}	15.01	Plastic
$HfSe_3$	42075	$P2_1/m$	1.07	0.46	3.28×10^{-2}	15.00	Plastic
ReS_2	81814	$P\bar{1}$	3.06	0.50	1.04×10^{-1}	14.74	Plastic
WTe_2	653168	$Pmn2_1$	2.53	0.60	1.04×10^{-1}	14.69	Plastic
HfS_3	42074	$P2_1/m$	1.10	0.42	3.17×10^{-2}	14.48	Plastic
ZrS_2	604434	$P\bar{3}m1$	1.73	0.43	5.33×10^{-2}	13.89	Plastic
$NbTe_2$	645529	$P\bar{3}m1$	1.86	0.63	8.77×10^{-2}	13.42	Plastic
$HfSe_2$	198541	$P\bar{3}m1$	1.69	0.44	5.59×10^{-2}	13.29	Plastic
TcS_2	81816	$P\bar{1}$	2.66	0.49	1.02×10^{-1}	12.87	Plastic
$ZrTe_3$	51004	$P2_1/m$	1.59	0.45	5.54×10^{-2}	12.82	Plastic
$TaSe_2$	26249	$P6_3mc$	3.26	0.60	1.55×10^{-1}	12.64	Plastic
SnS_2	651013	$P\bar{3}m1$	1.22	0.33	3.18×10^{-2}	12.53	Plastic
SiS_2	291212	$P2_1/c$	1.61	0.27	3.51×10^{-2}	12.15	Plastic
$MoTe_2$	14349	$P2_1/m$	2.45	0.61	1.25×10^{-1}	12.06	Plastic
In_2Se_3	4478	R3m	1.28	0.47	5.02×10^{-2}	11.97	Plastic
$SnSe_2$	651921	$P\bar{3}m1$	1.05	0.36	3.15×10^{-2}	11.86	Plastic
Hf_3Te_2	259226	I4/mmm	3.16	0.74	2.01×10^{-1}	11.71	Uncertain
Ho_2Te_5	639768	Cmcm	1.60	0.57	7.76×10^{-2}	11.63	Uncertain
TiS_2	601315	$P\bar{3}m1$	1.84	0.57	9.11×10^{-2}	11.45	Uncertain
$MoTe_2$	15431	$P2_1/m$	2.44	0.62	1.36×10^{-1}	11.12	Uncertain
$ZrSe_2$	652240	$P\bar{3}m1$	1.60	0.46	6.64×10^{-2}	11.07	Uncertain

Nd_2Te_5	646005	Cmcm	1.54	0.48	6.68×10^{-2}	10.95	Uncertain
$TiSe_2$	80091	$P\bar{3}m1$	1.72	0.64	1.09×10^{-1}	10.18	Uncertain
AuSe	73700	C2/m	1.83	0.77	1.39×10^{-1}	10.14	Uncertain
$CrSe_2$	626718	$P\bar{3}m1$	1.80	0.74	1.34×10^{-1}	9.93	Uncertain
FeSe	196301	P4/nmm	2.12	0.51	1.10×10^{-1}	9.92	Uncertain
$Ta_{21}Te_{13}$	91811	P6mm	3.86	0.49	1.94×10^{-1}	9.84	Uncertain
$DyTe_3$	630341	Cmcm	1.60	0.53	8.61×10^{-2}	9.81	Uncertain
Al_2Te_3	406353	$P2_1/c$	1.21	0.29	3.57×10^{-2}	9.79	Uncertain
$HoTe_3$	639770	Cmcm	1.58	0.53	8.58×10^{-2}	9.70	Uncertain
$ErTe_3$	631185	Cmcm	1.58	0.53	8.64×10^{-2}	9.67	Uncertain
$TiTe_2$	56011	$P\bar{3}m1$	1.59	0.74	1.22×10^{-1}	9.63	Uncertain
$LuTe_3$	642621	Cmcm	1.58	0.54	8.93×10^{-2}	9.54	Uncertain
$SmTe_3$	82602	Pbcm	1.54	0.50	8.28×10^{-2}	9.37	Uncertain
$NdTe_3$	646025	Cmcm	1.52	0.49	8.07×10^{-2}	9.29	Uncertain
Al_2Te_5	78941	C2/m	1.29	0.37	5.12×10^{-2}	9.27	Uncertain
$ZrTe_3$	42076	$P2_1/m$	1.11	0.45	5.42×10^{-2}	9.18	Uncertain
$NdTe_3$	170558	Cmcm	1.52	0.49	8.22×10^{-2}	9.17	Uncertain
$HfTe_2$	638959	$P\bar{3}m1$	1.54	0.55	9.51×10^{-2}	8.96	Uncertain
$CeTe_3$	170556	Cmcm	1.50	0.47	8.00×10^{-2}	8.89	Uncertain
$PrTe_3$	170557	Cmcm	1.49	0.48	8.09×10^{-2}	8.86	Uncertain
TaS_3	15251	$P2_1/m$	1.43	0.49	8.00×10^{-2}	8.69	Uncertain
$CoTe_2$	625401	$P\bar{3}m1$	1.74	1.04	2.11×10^{-1}	8.61	Uncertain
NbS_3	645316	$P2_1/m$	1.41	0.51	8.40×10^{-2}	8.53	Uncertain
$LaTe_3$	642056	Cmcm	1.47	0.47	8.53×10^{-2}	8.15	Uncertain
$TaSe_3$	651959	$P2_1/m$	1.24	0.50	7.84×10^{-2}	7.99	Uncertain
$NbSe_3$	645384	$P2_1/m$	1.23	0.49	7.64×10^{-2}	7.91	Uncertain
In_3Te_4	44655	$R\bar{3}m$	1.00	0.42	5.43×10^{-2}	7.75	Uncertain
$NbTe_2$	645536	C2/m	2.22	0.72	2.08×10^{-1}	7.74	Uncertain
$CrTe_3$	35266	$P2_1/c$	1.68	0.58	1.26×10^{-1}	7.69	Uncertain
CuTe	44993	Pmmn	1.30	0.51	8.57×10^{-2}	7.68	Uncertain

$ZrTe_2$	196804	$P\bar{3}m1$	1.52	0.62	1.26×10^{-1}	7.42	Uncertain
PtTe	41383	C2/m	2.11	0.98	2.82×10^{-1}	7.34	Uncertain
GeSe	41738	Pnma	1.20	0.56	9.12×10^{-2}	7.32	Brittle
$HfTe_3$	638958	$P2_1/m$	1.11	0.42	6.55×10^{-2}	7.13	Brittle
As_2Se_3	2600	$P2_1/c$	1.20	0.49	8.28×10^{-2}	7.07	Brittle
$BiSe_2$	194720	C2/m	1.21	0.76	1.32×10^{-1}	7.01	Brittle
PtTe	41370	$R\bar{3}m$	2.06	0.99	2.95×10^{-1}	6.89	Brittle
$IrTe_2$	189403	$P\bar{3}m1$	1.90	1.12	3.18×10^{-1}	6.66	Brittle
GeS_2	199200	$P4_2/nmc$	1.45	0.32	7.09×10^{-2}	6.56	Brittle
GeS	38165	Pnma	1.34	0.59	1.23×10^{-1}	6.43	Brittle
VTe_2	38369	C2/m	1.68	0.78	2.04×10^{-1}	6.39	Brittle
$NiTe_2$	142653	$P\bar{3}m1$	1.56	0.98	2.43×10^{-1}	6.27	Brittle
As_2S_3	15239	$P2_1/c$	1.33	0.43	9.12×10^{-2}	6.23	Brittle
Te_2Rh	26618	$P\bar{3}m1$	1.81	1.09	3.26×10^{-1}	6.09	Brittle
Bi_2Se_3	29345	$R\bar{3}m$	1.15	0.40	7.52×10^{-2}	6.07	Brittle
Bi_2Se_3	29345	$R\bar{3}m$	1.15	0.40	7.52×10^{-2}	6.07	Brittle
GeS	637785	Pnma	1.33	0.55	1.23×10^{-1}	5.97	Brittle
Pt_3Te4	133746	$R\bar{3}m$	1.97	1.01	3.35×10^{-1}	5.92	Brittle
$TaTe_2$	86141	C2/m	1.69	0.70	2.01×10^{-1}	5.88	Brittle
BiSe	617073	$P\bar{3}m1$	1.17	0.40	8.05×10^{-2}	5.81	Brittle
SbTe	20459	$P\bar{3}m1$	1.10	0.49	9.22×10^{-2}	5.80	Brittle
$PtSe_2$	649593	$P\bar{3}m1$	1.62	0.77	2.18×10^{-1}	5.73	Brittle
SnSe	651912	Pnma	0.95	0.57	9.51×10^{-2}	5.72	Brittle
SnSe	600595	Pnma	0.96	0.57	9.85×10^{-2}	5.55	Brittle
Te_2Pt	649747	$P\bar{3}m1$	1.68	0.90	2.77×10^{-1}	5.44	Brittle
$PdTe_2$	198013	$P\bar{3}m1$	1.62	1.06	3.25×10^{-1}	5.29	Brittle
GeS_2	1947	$P2_1/c$	1.51	0.22	6.45×10^{-2}	5.09	Brittle
GeSe	17660	Pnma	1.18	0.71	1.67×10^{-1}	5.03	Brittle
$ZrTe_5$	602282	Cmcm	1.01	0.39	7.92×10^{-2}	4.93	Brittle
BiTe	7742	$P\bar{3}m1$	1.07	0.46	9.92×10^{-2}	4.92	Brittle

BiTe	30525	$P\bar{3}m1$	1.07	0.46	9.96×10^{-2}	4.90	Brittle
In_2Te_3	640609	$R\bar{3}m$	0.98	0.52	1.03×10^{-1}	4.88	Brittle
$HfTe_5$	657474	Cmcm	1.00	0.39	7.90×10^{-2}	4.86	Brittle
Bi_2Te_3	44983	$R\bar{3}m$	1.04	0.45	9.61×10^{-2}	4.85	Brittle
Bi_2Te_3	199054	$R\bar{3}m$	1.04	0.45	9.64×10^{-2}	4.84	Brittle
Sb_2Te_3	20236	$R\bar{3}m$	1.01	0.48	1.00×10^{-1}	4.84	Brittle
As_2S_3	185819	P-1	1.35	0.36	1.01×10^{-1}	4.77	Brittle
SnS	24376	Pnma	1.05	0.58	1.35×10^{-1}	4.53	Brittle
BiTe	7744	$P\bar{3}m1$	0.96	1.00	2.11×10^{-1}	4.51	Brittle
SnS	651025	Pnma	1.04	0.58	1.35×10^{-1}	4.47	Brittle
In_2Te_5	640615	Cc	1.19	0.41	1.12×10^{-1}	4.32	Brittle
Sb_2Te	69557	$P\bar{3}m1$	1.12	0.64	1.72×10^{-1}	4.20	Brittle
Bi_4Te_3	23558	$R\bar{3}m$	1.05	0.61	1.53×10^{-1}	4.19	Brittle
GeTe	188458	R3m	0.94	1.13	2.53×10^{-1}	4.19	Brittle
GaTe	8249	C2/m	1.16	0.33	9.44×10^{-2}	3.99	Brittle
SnSe	50542	Pnma	0.95	0.57	1.42×10^{-1}	3.80	Brittle
GeTe	195457	R3m	0.99	1.06	2.76×10^{-1}	3.80	Brittle
$TmTe_3$	653102	Cmcm	0.53	0.53	7.67×10^{-2}	3.66	Brittle
$GdTe_3$	636467	Cmcm	0.52	0.52	7.60×10^{-2}	3.56	Brittle
$TbTe_3$	652952	Cmcm	0.52	0.52	7.54×10^{-2}	3.55	Brittle
YTe_3	653175	Cmcm	0.51	0.51	7.44×10^{-2}	3.54	Brittle
$GeSe_2$	614	$P2_1/c$	1.22	0.26	9.52×10^{-2}	3.37	Brittle
$CeTe_3$	622256	Cmcm	0.48	0.48	6.84×10^{-2}	3.31	Brittle
$PrTe_3$	649403	Cmcm	0.48	0.48	7.01×10^{-2}	3.29	Brittle
Dy_2Te_5	630329	Cmcm	0.50	0.50	8.21×10^{-2}	3.08	Brittle
Gd_2Te_5	636468	Cmcm	0.50	0.50	8.00×10^{-2}	3.08	Brittle
Ge_4Se_9	93460	$Pca2_1$	1.23	0.25	1.02×10^{-1}	3.06	Brittle

Supplementary Table 2.

High-throughput calculated ξ index (HT ξ), measured maximum bending strain; cleavage and slip energies, ξ, and $(E_{c,inter}E_{c,cross})/(E_{b,cross}E_{b,inter})$ by refined calculations for several 2D vdW crystals.

**Material**	**HT ξ ($J\ m^{-2}$)**	**Bending Strain (%)**	**Refined calculation**						
			$E_{c,inter}$ **($J\ m^{-2}$)**	$E_{b,inter}$ **($J\ m^{-2}$)**	$E_{c,cross}$ **($J\ m^{-2}$)**	**ξ ($J\ m^{-2}$)**	$E_{b,cross}$ **($J\ m^{-2}$)**	$E_{c,inter}E_{c,cross}/(E_{b,cross}E_{b,inter})$	**cross-layer slip system**
MoS_2	**40.27**	>20.00	0.56	5.44×10^{-2}	3.80	**39.12**	2.87	13.63	$(105)[\bar{5}01]$
					4.42	45.50	2.94	**15.48**	$(1\bar{1}0)[001]$
GaSe	**25.76**	>20.00	0.30	1.92×10^{-2}	1.60	**25.00**	1.22	20.49	$(11\bar{2})[201]$
					2.01	31.41	1.21	**25.96**	$(1\bar{1}0)[001]$
InSe	**21.42**	>20.00	0.30	1.90×10^{-2}	1.44	**22.74**	1.36	16.72	$(110)\ [001]$
					1.70	26.84	1.11	**24.18**	$(014)[\ 04\bar{1}]$
$SnSe_2$	**11.86**	>20.00	0.36	4.83×10^{-2}	1.05	**7.83**	0.64	**12.23**	$(\bar{1}02)[211]$
SnSe	**5.72**	0.66	0.63	1.12×10^{-1}	0.97	**5.46**	0.48	**11.38**	$(11\bar{1})[101]$
Bi_2Te_3	**4.85**	0.63	0.45	9.20×10^{-2}	1.04	**5.09**	0.80	6.36	$(018)\ [08\bar{1}]$
					1.09	5.33	0.45	**11.84**	$(\bar{1}05)[501]$
GeSe	**7.32**	3.60	0.61	1.07×10^{-1}	1.21	**6.90**	\	\	\
Bi_2Se_3	**6.07**	1.47	0.40	7.11×10^{-2}	1.15	**6.47**	\	\	\
Sb_2Te	**4.20**	0.62	0.65	1.75×10^{-1}	1.12	**4.16**	\	\	\
$NiTe_2$	**6.27**	0.62	1.04	2.78×10^{-1}	1.57	**5.87**	\	\	\
GaS	**26.43**	>20.00	0.29	2.05×10^{-2}	1.80	**25.46**	\	\	\
SnS_2	**17.94**	>20.00	0.33	3.51×10^{-2}	1.72	**16.17**	\	\	\
$MoTe_2$	**12.06**	>20.00	0.63	1.19×10^{-2}	2.45	**12.97**	\	\	\

Supplementary Table 3.

Potential cross-layer cleavage/slip systems for MoS_2 ($P6_3/mmc$), GaSe ($P6_3/mmc$), $SnSe_2$ ($P\bar{3}m1$), InSe ($P6_3/mmc$), SnSe (Pnma) and Bi_2Te_3 ($R\bar{3}m$).

Material	Plane	Cleavage energy ($J\ m^{-2}$)	Slip direction	Slip barrier energy ($J\ m^{-2}$)
MoS_2	(105)	3.80	[$\bar{5}$01]	2.87
	(0$\bar{1}$4)	4.17	[041]	3.17
	($\bar{2}$18)	4.29	[401]	3.43
	(1$\bar{1}$0)	4.42	[001]	2.94
GaSe	(11$\bar{2}$)	1.60	[111]	1.22
	(110)	1.64	[001]	1.41
	(114)	1.95	[$\bar{4}$01]	1.51
	(210)	1.95	[001]	1.32
	(1$\bar{1}$0)	2.01	[001]	1.21
$SnSe_2$	($\bar{1}$02)	1.05	[211]	0.64
	(023)	1.17	[03$\bar{2}$]	0.94
	(011)	1.28	[01$\bar{1}$]	0.84
	(11$\bar{1}$)	1.43	[101]	1.02
	(110)	1.47	[$\bar{1}$11]	0.91
InSe	(110)	1.44	[001]	1.36
	(114)	1.64	[22$\bar{1}$]]	1.32
	(120)	1.68	[001]	1.14
	(014)	1.70	[04$\bar{1}$]	1.11
	($\bar{1}$10)	1.72	[001]	1.14
SnSe[a]	(11$\bar{1}$)	0.97	[101]	0.48
	(110)	0.99	[001]	0.68
	(110)	1.01	[001]	0.61
Bi_2Te_3	(018)	1.04	[08$\bar{1}$]	0.80
	($\bar{1}$05)	1.09	[501]	0.45
	(13,1,0)	1.26	[$\overline{10}$,0,1]	0.70
	(110)	1.26	[001]	1.06

[a]Note: There are two slip/cleavage atomic planes parallel to (110) lattice plane with the inter-planar distances of 0.95 Å and 1.65 Å, respectively
