# Supplementary material for: High-throughput screening of 2D van der Waals crystals with plastic deformability
Source: Nat Commun. 2022 Dec 5;13:7491. doi: 10.1038/s41467-022-35229-x (PMC9723169; doi:10.1038/s41467-022-35229-x)
Supplement: Supplementary file 2 — Description of Additional Supplementary Files [file 41467_2022_35229_MOESM2_ESM.pdf]

File name: Supplementary Software 1

Description: Python codes for high-throughput computational screening in this work.
